# 

**Published:** 1838-07

**Authors:** 


### BRITISH AND FOREIGN

### MEDICAL REVIEW

OR

### QUARTERLY JOURNAL

OF

### PRACTICAL MEDICINE AND SURGERY

---

EDITED BY

JOHN FORBES M.D. F.R.S.

AND

JOHN CONOLLY M.D.

EDITORS OF THE CYCLOPÆDIA OF PRACTICAL MEDICINE

---

VOL. VI.

JULY—OCTOBER 1838

S.G.O.L. INDEXED

Surgeon Genl's Office LIBRARY. 34529 Washington, D.C.

LONDON

JOHN CHURCHILL PRINCES STREET SOHO

MDCCCXXXVIII

W
1
BR 34

LONDON:
PRINTED BY C. ADLARD, BARTHOLOMEW CLOSE.

### BOOKS RECEIVED FOR REVIEW.

ENGLISH.

1. A short Sketch of Animal Magnetism: intended to direct attention to the Propriety of practically examining the Question. By a Physician.—London, 1838. 8vo. pp. 63.

2. A Practical Treatise on Fractures. Illustrated by sixty Woodcuts. By E. F. Lonsdale, Surgeon, Demonstrator of Anatomy at the Middlesex Hospital School of Medicine.—London, 1838. 8vo. pp. 536. 16s.

3. Velpeau's Anatomy of Regions. Translated from the French, by Henry Hancock, Lecturer on Anatomy at the Westminster Hospital School of Medicine, &c.—London, 1838. 8vo. pp. 565. 16s.

4. Libamenta Praxeos Medicæ; or, a Manual of the Practice of Medicine, for the use of Students and junior Practitioners. By D. Spillan, M.D.—London, 1838. 48mo. pp. 192. 2s.

5. A Collection of Medical Formulæ, selected from the Writings of the most eminent Physicians. By D. Spillan, M.D.—London, 1838. 48mo. pp. 111. 1s.

6. The Final Report of the Committee of the Philadelphia Medical Society on the Construction of Instruments, and their Mode of Action in the radical Cure of Hernia, &c. By Heber Chase, M.D.—Philadelphia, 1837. 8vo. pp. 243.

7. The Philadelphia Practice of Midwifery. By C. D. Meigs, M.D., Lecturer on Midwifery, &c.—Philadelphia, 1838. 8vo. pp. 370.

8. Guy's Hospital Reports, No. VI. April, 1838. 8vo. pp. 267. 6s.

9. Manual of British Botany; in which the Orders and Genera are arranged and described according to the Natural System of De Candolle, &c. By D. C. Macreight, M.D. &c.—London, 1837. 12mo. pp. 296. 7s. 6d.

10. Remarks on a Case of Suicide published by Dr. P. D. Handyside. By J. R. Cormack, M.D.—Edinburgh, 1838. 8vo. pp. 16.

11. On the Differences of the Laws regulating Vital and Physical Phenomena. By W. B. Carpenter, M.R.C.S. &c. (From the Edinburgh Philosophical Journal.)—Edinburgh, 1838. 8vo. pp. 27.

12. Second Report of the New Lying-in Hospital, Dublin. By T. E. Beatty, M.D. Master of the Hospital.—Dublin, 1837. 8vo. pp. 43.

13. General View of the Proximate Causes of Diseases, organic and dynamic. By William Thomson, M.D. (From the Encyclopædia Britannica.) — Edinburgh, 1838. 4to. pp. 20.

14. The Responsibilities of Medical Students: a Sermon, preached in the Chapel of Guy's Hospital. By the Rev. F. Maurice, A.M.—London, 1838. 8vo. pp. 42.

15. The Medical Portrait Gallery, with Biographical Memoirs of the most celebrated Physicians, Surgeons, &c. who have contributed to the advancement of Medical Science. By T. J. Pettigrew, F.R.S. &c. —London, 1838. Nos. I. to III. Royal 8vo. 3s. each.

16. Experiments and Observations on the Gastric Juice, and the Physiology of Digestion. By W. Beaumont, M.D., Surgeon in the United States' Army. Reprinted, with Notes, by Andrew Combe, M.D. &c.—Edinburgh, 1838. 8vo. pp. 319. 7s.

17. Practical Observations on the Preservation of Health and the Prevention of Diseases; comprising the Author's Experience on the Disorders of Childhood and Old Age, on Scrofula, and on the Efficacy of Cathartic Medicines. By Sir Anthony Carlisle, F.R.S., President of the Royal College of Surgeons, &c.—London, 1838. 8vo. pp. 154. 8s.

18. A Series of Anatomical Sketches and Diagrams, with Descriptions and References. By T. Wormald, Assistant Surgeon, and A. M. M'Whinnie, Teacher of Practical Anatomy at St. Bartholomew's Hospital.—London, 1838. 4to. pp. 16; Five Plates. 4s.

19. On the Evidence of the occasional Contagious Propagation of Malignant Cholera. By J. Y. Simpson, M.D.—Edinburgh, 1838. 8vo. pp. 54. (From the Edinburgh Journal.)

20. A Dissertation on the Causes and Effects of Disease, considered in reference to the Moral Constitution of Man. By Henry Clark Barlow, M.D.—Edinburgh, 1837. 8vo. pp. 79. 3s.

21. On the Improvement of Medicine. The Oration delivered before the Medical Society of London, at their Sixty-fifth Anniversary, March 8th, 1838. By Theoph. Thompson, M.D.—London, 1838. 8vo. pp. 23.

22. Buxton and its Waters: an Analytical Account of their Medical Properties and general Effects. By W. H. Robertson, M.D.—London, 1838. 12mo. pp. 147. 2s. 6d.

23. Fifth Annual Report of the Trustees of the [Massachusetts] State Lunatic Hospital, at Worcester.—Boston, 1838. 8vo. pp. 71.

24. Outlines of Physiology, designed for the use of the higher Classes in common Schools. By G. Hayward, M.D., Professor of Surgery in Harvard University. Second Edition. — Boston, 1838. 12mo. pp. 222.

25. Germany: the Spirit of her History, Literature, Social Condition, and Natural Economy, &c. By Bisset Hawkins, M.D. F.R.S. &c.—London, 1838. 8vo. pp. 475. 10s. 6d.

26. A Practical Compendium of the Materia Medica; with numerous Formulæ adapted for the Treatment of the Diseases of Infancy and Childhood By Alexander Ure, M.D.—London, 1838. 12mo. pp. 221. 6s.

27. Third Report of the Inspectors appointed to visit the different Prisons of Great Britain. Southern and Western District. Presented to both Houses of Parliament, by command of her Majesty. (From Dr. Bisset Hawkins.) — London, 1838. Folio, pp. 120.

28. Practical and Experimental Chemistry, adapted to Arts and Manufactures. By E. Mitscherlich, Professor of Chemistry in the University of Berlin. Translated by S. L. Hammick, M.D., Fellow of the Royal College of Physicians, &c.—London, 1838. 8vo. pp. 316. 10s. 6d.

29. Animal Magnetism and Homœopathy. By Ed. Lee, M.R.C.S. &c. Second Edition, considerably enlarged.—London, 1838. 8vo. pp. 98. 3s. 6d.

30. The Narrative of a Recovery from Tic Douloureux. By the Reverend C. E. Hutchinson, Vicar of Firle, Sussex.—London, 1838. 8vo. pp. 43.

31. A Dictionary of Practical Medicine. By James Copland, M.D. F.R.S. &c. Part V. Heart–Infection.—London, 1838. 4s.6d.

32. Elements of Physiology. By J, Müller, M.D. &c. Translated, with Notes, by W. Baly, M.D. Part III.—London, 1838. 8vo. 4s. 6d.

33. Transactions of the Provincial Medical and Surgical Association. Vol. VI. 8vo. pp. 621. 16s.

FOREIGN.

1. Medicinisch-psychologisches Gutachten über die Verurtheilung des Lieutenants Emile de la Roncière vor den Assisen in Paris, im Jahre, 1835. Von C. C. Matthæi, M.D.—Hannover, 1836. 8vo. pp. 84.

2. Ein Fragment aus dem Verhältnisse Marienbads zu seinen Lebendigen und Todten, von 1833 und 1834. Von Dr. J. C. Heidler.—Prag. 1837. 12mo. pp. 35.

3. Alle Gründe für den neuen Ruf von Marienbad. Von Dr. C. J. Heidler.—Prag. 1837. 12mo. pp. 52.

4. Vier Abbildungen des Schädels der Simia Satyrus, von Verschiedenem Alter, zur Aufklärung der Fabel vom Oran Utan herausgeben von Dr. C. F. Heusinger.—Marburg, 1838. 4to. pp. 44.

5. Memorie sul Cholera-Morbus, publicate per cura della Società Medico-chirurgica di Bologna. Fasc. 3°.—Bologna, 1836. 8vo.

6. Heilungen durch Animalischen Magnatismus bewirkt. Herausgegeben von Dr. J. Bork. — Würzburg, 1837. 8vo. pp. 97.

7. Der Mineralische Magnatismus als heilmittel. Von Dr. Fickel. — Leipzig, 1836. 8vo. pp. 32.

8. Resumé de Notions Medico-topographiques sur les Sources Salines de Stara-Roussa. — St. Petersbourg, 1837. 8vo. pp. 32.

9. Ephebi ad Nobilem juventutem in re militari informandam primitus stabiliti Diatriba physico-iatrica, elaborata medico primario ejusdem instituti Martino Solsky. —Petropoli, 1837. 8vo. pp. 104.

10. Freihefte für wissenschaftliche Kritik und Antikritik in der Natur- und Heilkunde. Herausgegeben von L. A. Kraus, M.D. — Göttingen, 1837. 8vo. pp. 150.

11. Die Lehre vom Mechanismus der Geburt nebst Beiträgen zur Geschichte derselben. Von H. F. Naegele, M.D., Privatdocenten an der Universität Heidelberg. —Mainz, 1838. 8vo. pp. 243.

12. Ausführliche Encyclopädie der gesammten Staatsarzneikunde. Herausgegeben von G. F. Most, M.D. &c. Erstes Heft. Aal—Arzt.—Leipzig, 1838. 8vo. pp. 192.

13. C. Pruys van der Hoeven, de Arte Medica, libri duo ad Tirones. Liber Primus. Pars Prior. De Inflammationibus. —Lugd. Batav. 1838. 8vo. pp. 559.

14. Forelæsninger over den legale Medicin af Dr. Michael Skjelderup, Professor i Lægevidenskaben ved det Kongl. Frederiks Universitat, &c.— Christiania, 1838. 8vo. pp. 212.

15. Deceptatio Medico Inauguralis de Sanguine quam pro gradu Doctoratus in Academia Lugduno - Batava, defendet Gulielmus Munk, e Collegio Universitate Londinensi, &c.— Lugduni Batavorum, 1837. 8vo. pp. 28.

